# Thermal regimes of Rocky Mountain lakes warm with climate change

**DOI:** 10.1371/journal.pone.0179498

**Published:** 2017-07-06

**Authors:** James J. Roberts, Kurt D. Fausch, Travis S. Schmidt, David M. Walters

**Affiliations:** 1 Colorado Water Science Center, U.S. Geological Survey, Fort Collins, Colorado, United States of America; 2 Fort Collins Science Center, U.S. Geological Survey, Fort Collins, Colorado, United States of America; 3 Department of Fish, Wildlife, and Conservation Biology, Colorado State University, Fort Collins, Colorado, United States of America; Universidade de Aveiro, PORTUGAL

## Abstract

Anthropogenic climate change is causing a wide range of stresses in aquatic ecosystems, primarily through warming thermal conditions. Lakes, in response to these changes, are experiencing increases in both summer temperatures and ice-free days. We used continuous records of lake surface temperature and air temperature to create statistical models of daily mean lake surface temperature to assess thermal changes in mountain lakes. These models were combined with downscaled climate projections to predict future thermal conditions for 27 high-elevation lakes in the southern Rocky Mountains. The models predict a 0.25°C·decade^-1^ increase in mean annual lake surface temperature through the 2080s, which is greater than warming rates of streams in this region. Most striking is that on average, ice-free days are predicted to increase by 5.9 days ·decade^-1^, and summer mean lake surface temperature is predicted to increase by 0.47°C·decade^-1^. Both could profoundly alter the length of the growing season and potentially change the structure and function of mountain lake ecosystems. These results highlight the changes expected of mountain lakes and stress the importance of incorporating climate-related adaptive strategies in the development of resource management plans.

## Introduction

Aquatic systems are being altered by anthropogenically driven changes in climatic conditions [1–5]. Changes in climate are causing a wide range of stresses in aquatic systems, and a particularly important one is increasing water temperature, which has been documented in streams and lakes worldwide [5–7]. Lakes are important sentinels of climate change [8,9] and the routes to change in lakes include three primary climate forcing pathways: temperature, precipitation, and incident solar radiation [10]. Rising water temperatures in lakes is a particularly important change because lakes have warmed faster than atmospheric temperatures in some cases [11,12]. Worldwide, the summer surface temperatures of lakes are increasing at an average rate of 0.34°C ·decade^-1^ [5]. These high rates of temperature rise are likely to have significant effects on lakes because thermal regime has a strong influence on the structure and function of lentic ecosystems [13–15]. Specific lake processes that are susceptible to thermal changes include plankton bloom phenology [16], harmful algal blooms [17], depletion of hypolimnetic dissolved oxygen concentrations [18], and thermal suitability for vertebrates [19,20].

Although changing thermal conditions are predicted for lakes of all sizes and types, the influence of a changing climate on abiotic and biotic conditions of high-elevation mountain lakes remains poorly studied. Small lakes are common features of mountainous landscapes, where headwater systems function as important drivers of watershed processes [21]. One region where headwater lakes are especially important to biogeochemical and hydrologic processes is the southern Rocky Mountains (SRM; [22,23]). Within the SRM, a semi-arid portion of western North America, mountain lakes and corresponding headwater networks influence important water management decisions through their storage and release of snowmelt, an important hydrologic driver of streams, rivers, and reservoirs [24–27]. Air temperatures in the SRM are warming, although these trends are mostly confined to the summer and fall seasons [28], and the magnitude of these changes are elevation dependent [29]. Therefore, understanding how high-elevation mountain lakes respond to climate change is important for water management in the SRM and other regions where anthropogenic needs depend on mountain hydrology.

Mountain lakes and associated headwater regions also provide critical refuge habitats for native species throughout the SRM, many of which are imperiled (e.g., Cutthroat Trout *Oncorhynchus clarkii* spp. and several amphibians; [30–32]). Various aspects of climate change, including warming temperatures, have the potential to alter the unique habitats that these lakes provide. However, the influence of changing climatic conditions on mountain lakes in the SRM has been previously addressed mostly through broad scale generic modeling of North American lakes (i.e., [33–35]) and a limited number of empirically-based studies of individual mountain lakes [28,36]. Thus, uncertainty remains regarding how the thermal conditions of SRM lakes have changed in the past, the magnitude of likely future changes, and the potential landscape-level consequences of these changes for lake-dependent species and ecosystem processes.

Here we use continuous records of surface water temperature for individual lakes and weather station measurements of air temperature to create statistical models of daily mean lake surface temperature. The fine-scale temporal resolution of our predictions facilitates detailed analyses of SRM lake thermal regimes. We combine these statistical models with dynamically downscaled regional climate model predictions to forecast lake surface water temperature trends over the next 70 years, and test the hypothesis that mountain lakes in the SRM are warming in response to climate change. Our model results provide information and models crucial to the creation of climate-smart management and conservation plans for mountain lakes and their biota.

## Materials and methods

### Temperature data

We compiled data for 27 lakes, the majority of which are in Rocky Mountain National Park (S1 Table), all of which had continuous records at least 2 years long (range: 2 to 15 years). Temperature was recorded every 60 minutes for the entire duration of each continuous lake record. We performed an initial quality check of these data using protocols established for stream temperature data [37]. Only georeferenced data collected within the top 3 meters of the water column or located within the first 250m of the lake inlet or outlet stream were included in our analysis. Hydrogeomorphic site characteristics for each lake (S1 Table) were collected during logger deployment (maximum depth and elevation) and downloaded from the National Hydrography Dataset Plus Version 2 (surface area and cumulative drainage area; NHDPlusV2; http://www.horizon-systems.com/nhdplus/). To use the data collected in Rocky Mountain National Park we received approval for this project from the park and we performed this research under a Scientific Research and Collection Permit (#ROMO-2013-SCI-0009).

We acquired air temperature records from the snow telemetry (SNOTEL) network and created a database of the 21 SRM SNOTEL sites nearest the lakes with temperature data. Daily summaries of average air temperature (°C) were downloaded from the SNOTEL webportal (http://www.wcc.nrcs.usda.gov/snow/) and used to calculate the 7-day running mean of daily air temperature (hereafter, mean weekly temperature). We used this metric as the independent variable to create water temperature models of daily mean lake surface temperatures for each lake and, in turn, to predict past and future lake temperatures.

### Model development

**Fig 1:**
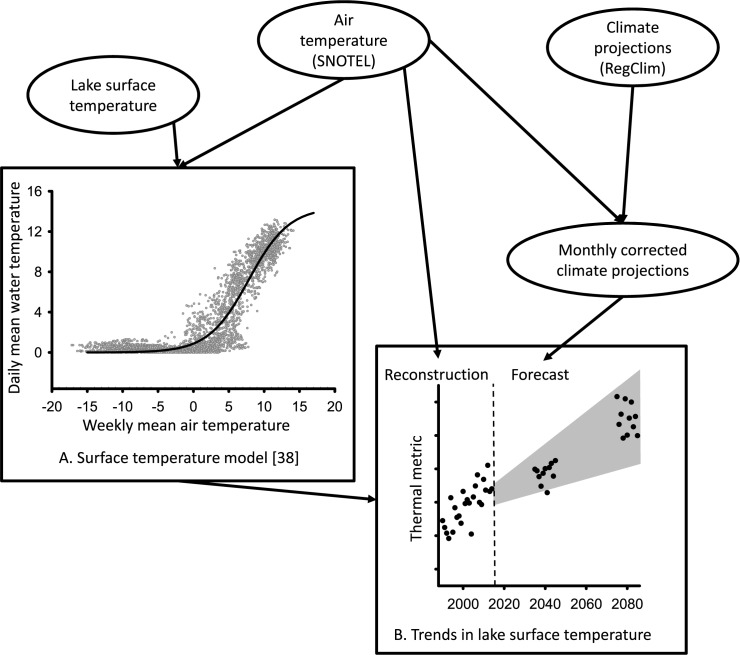
Lake temperature modeling process. Schematic of the process used to create models of lake surface temperature and predicted future thermal conditions of southern Rocky Mountain lakes. Input variables are shown in ovals and the model of surface temperature and model output are in rectangles. Thermal metrics include the warmest 30-day running mean of daily mean water temperature (M30AT), the mean annual surface temperature (MAT), the mean summer surface water temperature (MST), and number of ice-free days (IFD). The gray polygon in B indicates upper and lower bounds of the predictions derived from uncertainty analysis (see Materials and Methods).

We created a separate model of daily mean lake surface temperature for each lake. We used air temperature from the nearest SNOTEL site (via Euclidian distance) and selected data to match the period of lake temperature record. We used mean weekly air temperature as a predictor of daily mean lake surface temperature (Fig 1A), and created predictive models using non-linear logistic regression [38,39]. This modeled relationship represents how air temperature and lake surface temperature co-vary, with extreme low air temperature rarely driving lake temperature below 0°C and an asymptotic upper limit of lake temperature controlled by evaporative cooling [39]. Model parameters were fit using a non-linear least-squares loss function and the Gauss-Newton algorithm. All models were fit using SYSTAT 12 software (Chicago, IL). We evaluated model fit using the Nash Sutcliffe Coefficient (NSC; [40]) where a perfect model fit receives a value of 1 and a poor model fit can receive negative values. The NSC is a more effective measure than the Root Mean Squared Error for evaluating the fit of non-linear models [39].

### Lake temperature predictions

#### Reconstructing lake temperature

We employed these non-linear models to predict past lake surface temperature (reconstructing) using the entire air temperature time series (1986–2014) from the SNOTEL sites. These reconstructions serve as baseline conditions for assessing projected future thermal conditions for each lake (Fig 1B).

#### Projections of lake temperature

We used the model developed for each lake to project future thermal conditions from dynamically downscaled climate projections [41]. The spatial resolution of these climate projections is 15x5-km grid cells, and projections are available for short-term (2035–2045) and long-term (2075–2085) time horizons. We used daily output of surface air temperature from the PSU/OSU GENMOM (GNM; [42]) and MPI ECHAM5 (ECH; [43]) climate models, which were dynamically downscaled using a regional climate model (RegCm3; [41]) under the A2 emission scenario from the IPCC AR4 (International Panel of Climate Change) which assumes a medium-to-high emissions future [44]. The A2 emission scenario is most similar to the RCP 8.5 carbon scenario used in the newest IPCC AR5 efforts [45]. Moreover, the GNM and ECH climate models were the only two models from Hostetler et al. [41] that provided daily output suitable for our analysis. We compared daily air temperatures measured at each SNOTEL site used in this study with temperatures predicted from each climate model within the overlaying grid cell of each SNOTEL site for 2010–2012 (the only overlapping years available) and created an average monthly correction factor. We applied this additive monthly correction factor to all climate projections before predicting lake surface temperature (Fig 1B). These future air temperatures were summarized as weekly mean temperatures and used as input into each lake-specific model to predict future daily surface water temperature.

We performed an uncertainty analysis using each of the two climate models to create a new data-series of highest and lowest predicted daily mean air temperatures, since the highest and lowest value for a given day were not consistently associated with one model. Using this dataset of predicted high and low daily air temperature we calculated mean weekly high and low air temperatures. We then used these to predict high and low daily surface water temperature for each lake, which defined the upper and lower bounds of our predictions (Fig 1B).

### Lake thermal metrics

We used the daily values of lake surface temperature reconstructed and predicted from forecasting to calculate four lake thermal metrics (Fig 1). The first is a measure of fish habitat quality [31] and represents the warmest 30-day running mean of daily mean water temperature (M30AT). The other three metrics are the mean annual surface water temperature (MAT), the mean summer surface water temperature (mean for June-August of each year; MST), and the number of ice-free days (sum of days with a daily mean lake surface temperature ≥4°C for each year; IFD). This estimate of IFD is based on lake hydrodynamics and assumes that when the lake surface water is 4°C (i.e., the value at which liquid water is most dense) the entire water column is iso-thermal with no ice cover [46]. We summarize these thermal metrics at three different time horizons: current (2004–2014), 2040s (2035–2045), and 2080s (2075–2085). We also used the annual predictions of these metrics to examine trends (i.e., rates of change) in our thermal metrics from 1986–2002 (depending on the record of the closest SNOTEL site) through 2085 using linear regression (α = 0.05), with year as the independent variable. We used the slopes from these linear regressions to represent the rate of change for each temperature metric, and present all results as rates of change ·decade^-1^. These statistical analyses were performed using SYSTAT 12 (Chicago, IL) software.

#### Fish thermal habitat quality

We assessed the thermal habitat quality for native fishes (Cutthroat Trout) in these mountain lakes using eco-physiological thresholds defined in the literature. Roberts et al. [31] related M30AT to growth and recruitment of Cutthroat Trout in SRM streams and defined five eco-physiological states which are directly relevant to thermal habitat quality of mountain lakes for these fish. Briefly, a M30AT below 8.0°C is too cold for growth and survival of young trout. Temperatures in the 8.0–9.0°C range are cold enough to restrict the survival of young trout up to age-1. However, higher temperatures (9.1–18.0°C) are optimal for growth and recruitment of trout, while even warmer temperatures (18.1–19.9°C) can reduce growth. Finally, temperatures in excess of 20.0°C limit or stop growth of Cutthroat Trout [31]. We use these eco-physiological thresholds to evaluate the fish thermal habitat quality in these 27 mountain lakes and examine changes through the 2080s.

## Results

### Model development

The models of surface temperature for each of the 27 lakes had an average NSC value of 0.83, although the model parameters and fit varied among lakes (NSC range 0.68–0.91; S2 Table). The average model parameter for maximum temperature (α) was 20.1°C, the average parameter for minimum temperature (μ) was 0.0°C, the average *γ* (i.e., parameter defining slope between α and μ) was 0.3, and the average β (i.e., the parameter defining inflection point) was 12.0°C (S2 Table; see [38] for more details of model equation and parameters). The variation of these parameter values among water bodies supports our decision to fit individual models for each lake.

### Lake temperature predictions

**Fig 2:**
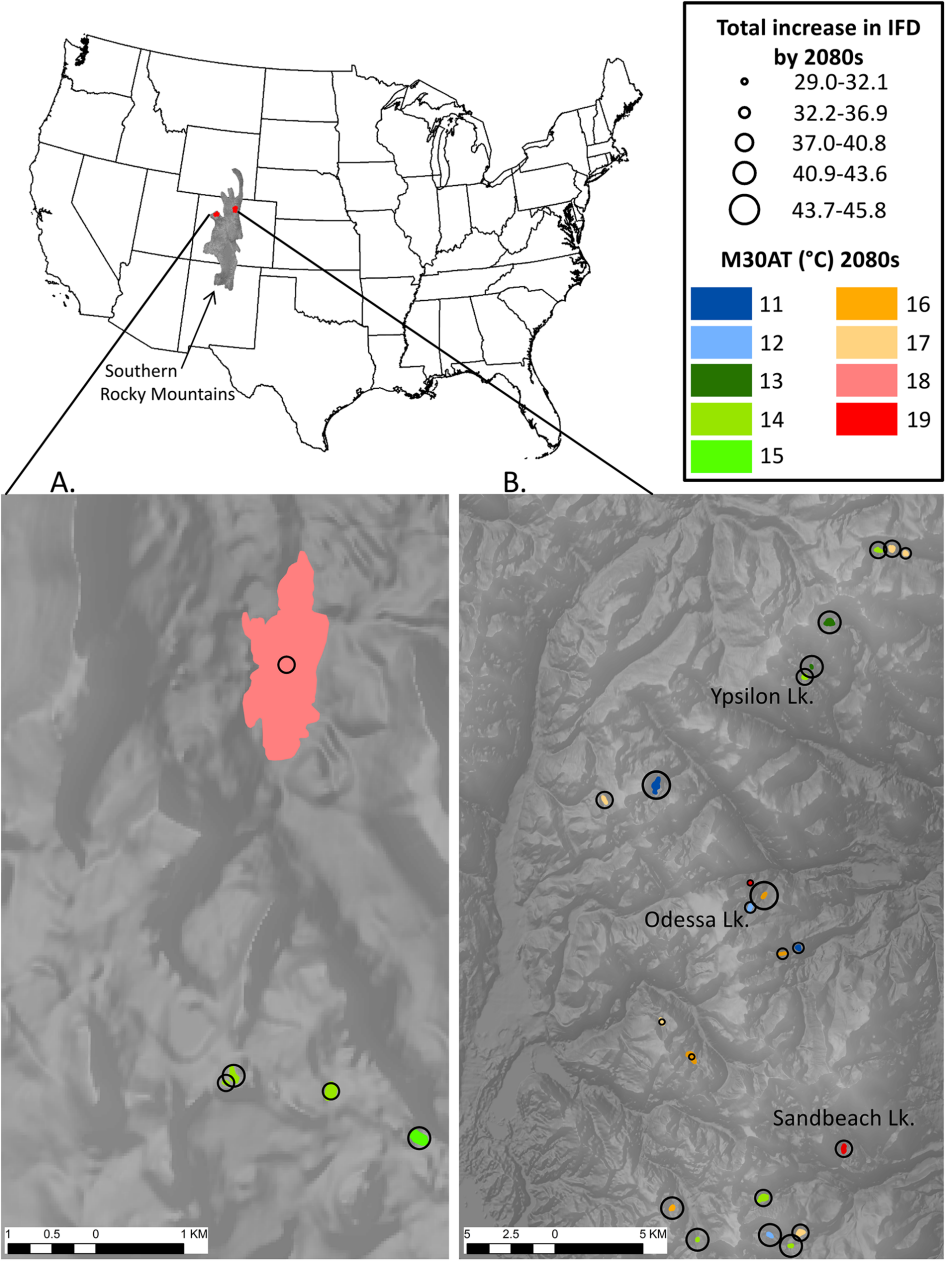
Future thermal conditions of southern Rocky Mountain lakes. Location, relative size, and juxtaposition of each study lake. Maps show portions of the Flat Tops Wilderness (A.) and Rocky Mountain National Park (B.) within the Southern Rocky Mountains. Model predictions of M30AT in the 2080s are shown in color for each of the 27 lakes, and the predicted increase in the number of ice-free days (IFD) over seven decades (circles). The three lakes that are labeled were used to present our uncertainty analysis (see Fig 3).

We used the measured and predicted air temperature in our models of lake temperature to predict daily lake surface temperature during past, current, and future periods for each of the 27 lakes, the slopes at all lakes are significant at α = 0.05 (Fig 1B). The current (2004–2014) MAT among lakes of 3.9°C (range 2.2–6.4) increased to 5.5°C (3.4–7.9) by the 2080s. On average, MAT increased 0.25°C ·decade^-1^from 1986 through 2085 resulting in a 41% mean increase over current conditions by the 2080s (Table 1). In contrast, the magnitude of change in MST was roughly double that predicted for MAT. The current MST of 9.8°C (5.9–13.7) increased to 12.7°C (8.3–16.8) by the 2080s, a 30% mean increase, resulting in an overall rate of increase of 0.47°C ·decade^-1^ (Table 1). Large increases in IFD are also predicted. The current mean IFD of 128 days (86–184) increased to 165 days (131–217) IFD by the 2080s. The mean overall rate of change for IFD was 5.9 days ·decade^-1^, resulting in 37 more days without ice cover by the 2080s compared to current conditions (2004–2014), a 29% increase (Table 1; Fig 2). Finally, the thermal suitability of SRM lakes for native fishes also changed. The current M30AT for these lakes is 12.1°C on average (7.8–15.8; S1 Table), whereas the mean M30AT predicted for the 2080s is 15.0°C, an increase of 2.9°C or 24% from current conditions (Table 1).

**Table 1:** Trends in thermal conditions for southern Rocky Mountain lakes. Predicted change in lake surface temperature metrics for 27 southern Rocky Mountain lakes. Metrics were calculated using the lake-specific non-linear models of daily mean lake surface temperature, mean weekly air temperatures form SNOTEL sites, and future mean weekly air temperature predicted from climate models [41]. Rates of change ·decade^-1^ were calculated by linear regression using annual measures of each metric as a function of year. Upper and lower bounds of these predictions from our uncertainty analysis are shown in parentheses.

Lake	Increase per decade			M30AT 2080s (°C; i.e., Warmest 30days)
	Mean summer (°C; MST)	Mean annual (°C; MAT)	Ice free days (IFD)	
Adams Lake	0.52 (0.32, 0.72)	0.27 (0.14, 0.40)	6.4 (3.9, 8.5)	16.1 (14.6, 16.9)
Arrowhead Lake	0.36 (0.15, 0.43)	0.17 (0.09, 0.25)	6.7 (3.9, 8.3)	10.7 (09.3, 11.3)
Bear Lake	0.29 (0.17, 0.38)	0.17 (0.08, 0.25)	5.2 (3.3, 7.1)	11.7 (10.8, 12.1)
Big Cow Lake	0.54 (0.34, 0.72)	0.23 (0.12, 0.31)	6.2 (4.0, 7.5)	13.7 (12.1, 14.5)
Bluebird Lake	0.60 (0.48, 0.71)	0.27 (0.18, 0.36)	6.1 (4.1, 7.1)	13.7 (13.0, 14.1)
Boundary Lake	0.47 (0.31, 0.64)	0.24 (0.13, 0.35)	6.4 (3.9, 8.4)	14.2 (12.9, 14.8)
Caddis Lake	0.42 (0.25, 0.58)	0.21 (0.11, 0.30)	6.5 (4.2, 8.2)	12.7 (11.2, 13.5)
Crystal Lake	0.44 (0.27, 0.60)	0.22 (0.12, 0.32)	6.4 (4.1, 8.2)	13.4 (12.1, 14.1)
Dream Lake	0.43 (0.27, 0.56)	0.25 (0.13, 0.35)	5.3 (3.2, 7.3)	15.8 (14.8, 16.3)
Fern Lake	0.59 (0.37, 0.80)	0.34 (0.21, 0.47)	6.8 (4.1, 9.2)	16.2 (14.7, 17.4)
Gem Lake	0.50 (0.34, 0.67)	0.23 (0.14, 0.33)	6.0 (4.3, 7.2)	14.3 (13.0, 14.9)
Jewel Lake	0.64 (0.39, 0.83)	0.26 (0.15, 0.36)	6.3 (4.1, 7.4)	15.4 (13.5, 16.4)
Lake Husted	0.55 (0.34, 0.75)	0.27 (0.14, 0.39)	5.9 (3.4, 7.9)	16.6 (15.0, 17.4)
Lake Louise	0.46 (0.29, 0.63)	0.22 (0.11, 0.33)	5.9 (3.4, 7.7)	13.8 (12.5, 14.5)
Lake Nanita	0.41 (0.26, 0.56)	0.24 (0.12, 0.36)	4.8 (2.8, 7.1)	16.3 (15.1, 17.0)
Little Cow Lake	0.55 (0.35, 0.74)	0.23 (0.13, 0.33)	5.9 (4.0, 7.3)	14.3 (12.7, 15.1)
Lost Lake	0.48 (0.31, 0.66)	0.26 (0.13, 0.39)	5.4 (3.1, 7.7)	16.7 (15.5, 17.4)
Lower Hutcheson Lake	0.45 (0.28, 0.62)	0.23 (0.12, 0.34)	6.5 (3.9, 8.3)	13.7 (12.4, 14.3)
Odessa Lake	0.33 (0.21, 0.44)	0.19 (0.10, 0.27)	5.5 (3.6, 7.4)	12.4 (11.5, 12.9)
Pear Lake	0.56 (0.37, 0.74)	0.29 (0.16, 0.43)	6.0 (3.5, 8.4)	17.3 (16.0, 18.0)
Pettingell Lake	0.32 (0.23, 0.42)	0.25 (0.13, 0.37)	4.4 (1.5, 7.1)	17.3 (16.7, 17.6)
Sandbeach Lake	0.57 (0.37, 0.77)	0.33 (0.18, 0.48)	5.7 (3.2, 8.2)	19.5 (18.1, 20.2)
Spruce Lake	0.49 (0.31, 0.68)	0.28 (0.14, 0.42)	4.5 (2.0, 6.9)	19.4 (17.8, 20.3)
Timber Lake	0.55 (0.36, 0.78)	0.26 (0.14, 0.39)	5.9 (3.6, 7.7)	17.1 (15.4, 18.0)
Trappers lake	0.43 (0.28, 0.55)	0.28 (0.16, 0.39)	5.8 (2.3, 8.3)	17.6 (15.5, 18.8)
Upper Hutcheson Lake	0.41 (0.25, 0.57)	0.21 (0.11, 0.31)	6.4 (3.8, 8.3)	12.2 (10.1, 14.4)
Ypsilon Lake	0.42 (0.26, 0.57)	0.23 (0.12, 0.34)	5.9 (3.7, 7.9)	14.0 (12.2, 15.8)
**Mean (SE)**	**0.47 (0.02)**	**0.25 (0.01)**	**5.9 (0.1)**	**15.0 (0.4)**

**Fig 3:**
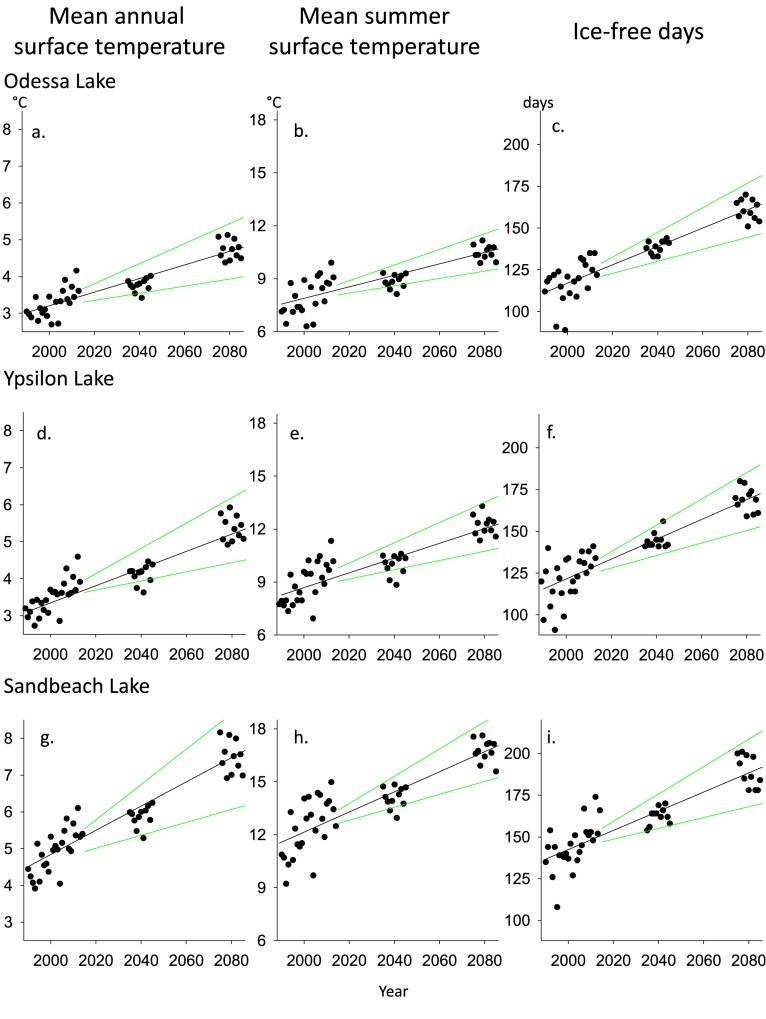
Trends in southern Rocky Mountain lake thermal regimes. Example plots of thermal change (1988–2085) and the uncertainty analysis for these rates of change in three thermal metrics for three representative lakes. These lakes span the range of relative thermal regimes observed which include cold (Odessa Lake; a-c), cool (Ypsilon Lake; d-f), and warm (Sandbeach Lake; g-i). Thermal metrics presented are the mean annual lake surface temperature (a,d,g), the mean summer lake surface temperature (b,e,h), and number of ice-free days (c,f,i). Green trend lines represent the upper and lower bounds of future thermal trends. These bounds are calculated using the highest and lowest projected mean weekly air temperatures from both climate models combined.

The uncertainty analysis showed that both the upper and lower bounds for all the thermal metrics are predicted to increase for all the lakes through the 2080s (Table 1; Fig 3). However, with time, the spread of the upper and lower bounds also increased about the mean trends for these three thermal metrics. The variation in the trend was greatest for MAT, for which the upper and lower bounds averaged ±45% lower and higher (0.13–0.36, respectively), than the mean rate for all the lakes of 0.25°C ·decade^-1^. Variation was less for IFD, for which the average bounds were ±36% lower and higher (3.51–7.79) than the mean rate of 5.9 days ·decade^-1^, and even less for MST, for which the average bounds were ±35% lower and higher (0.30–0.63) than the mean rate of 0.47°C ·decade^-1^. Across the gradient of thermal conditions, the thermal metrics of a representative warm lake (Sandbeach Lake) increased at greater rates and were more variable than a cool (Ypsilon Lake) or cold (Odessa Lake) lake.

#### Fish thermal habitat quality

Current mean M30AT for these 27 lakes is 12.1°C, on average, which is considered optimal [31] for recruitment and growth of native Cutthroat Trout (S1 Table). However, Arrowhead Lake is currently too cold for young fish survival and Caddis Lake is currently cold enough to decrease the survival of young fish (S1 Table). By the 2080s the models predict that none of these 27 lakes will be too cold for growth and survival of young fish, although two will be too warm. The models predict a mean M30AT of 15.0°C for the 27 lakes in the 2080s, which is considered optimal for growth and survival. Nevertheless, two individual lakes, Spruce Lake and Sandbeach Lake will be warm enough to reduce the growth rate of adult cutthroat trout by the 2080s (Table 1).

## Discussion

The surface temperature and IFD of high-elevation lakes in the SRM have already increased with warming climatic conditions, and are projected to increase more in the coming decades. The magnitude of these changes are greater than reported trends for lakes worldwide. For example, the predicted MST increase of 0.47°C ·decade^-1^ is more than a 0.10°C greater than the predicted increase of 0.34°C ·decade^-1^ for nighttime summer surface temperature measured for lakes worldwide [5]. However, our results indicate that temperature increases are unlikely to be great enough by the 2080s to make most of these lakes thermally unsuitable for native top predators (i.e., Cutthroat Trout) in these systems. Nevertheless, the magnitude of these changes is substantial. For example, the 30% and 41% predicted increases in MST and MAT, respectively, are likely to have unexpected consequences for abiotic characteristics, biogeochemical processes, and trophic dynamics of these important SRM headwater habitats, and hold the potential to cause ecological surprises [47,48] in the food webs of these mountain lakes.

The most striking thermal change for these high-elevation lakes is the 29% increase (i.e., a mean increase of 37 days) in IFD by the 2080s. Increasing length of the ice-free season has been observed and predicted for lakes worldwide, based on long records of lake and river surface freeze and thaw dates [49,50]. For example, a comprehensive assessment of ice cover for freshwater systems in the northern hemisphere observed (1822–1995) a 1.7 day decade ^-1^ increase in ice free days in 11 lakes (a subset of the water bodies included in the study; [49]). Our forecasted average increase in ice free days (5.9 days ·decade^-1^) is much greater than that observed in the recent past; nevertheless, the higher rates we predicted are supported by other models of future increases, such as an increase of 10–26 ice-free days in Canadian lakes by 2055 [51]. Another study using models of generic lakes across the contiguous United States predicted that a scenario where atmospheric CO_2_ concentrations doubled (no time period specified) from past levels (i.e., 1961–1979) could result in 90 more ice free days in lakes, depending on location [34]. An empirical study of one watershed in the SRM also supports our results, reporting that ice is thawing 2.1 days ·decade^-1^ earlier (average decadal rate, 1981–2014; [36]), which is similar to half (2.95 days ·decade^-1^) the change we predict for IFD. These changes in length of IFD are also likely to influence important ecological processes occurring during ice-cover, processes that are understudied and are important drivers of the ecosystem functions in temperate lakes [52]. Our study is the first to model the change in IFD for relatively cold, high-elevation lakes, and shows that these lakes will also lose ice at a drastic rate, similar to those predicted for lakes at lower elevations.

Our models predict that these lakes will warm at a faster rate than streams and rivers within the Rocky Mountain region. The MST in streams in the upper Columbia River drainage (Northern Rocky Mountains) is predicted to increase 2.1°C by the 2080s [53], substantially lower than our model predicted MST increase of 2.9°C. Likewise, we predict that lake M30AT will increase 2.9°C by the 2080s, nearly three-fold higher than the increase of 1.1°C predicted for high-elevation streams in the upper Colorado River basin [31]. The greater increase in lake temperatures when compared to streams and rivers in the SRM underscores the need to determine which thermal properties are driving ecosystem processes in these high-elevation lakes, to allow a better understanding of the ecological consequences of these thermal changes.

Although temperature itself is a dominant driver of biological processes [13], the timing and seasonal fluctuations of water temperature are likely as important, or more important. Although the predicted average summer temperatures (M30AT) are unlikely to have acute effects, such as extirpation, for many taxa in SRM lakes, the potential chronic effects of changes in thermal regime may greatly influence the physical habitats of these biota and ecosystem processes that support them. For example, the warming surface water temperatures are unlikely to influence hypolimnetic temperatures [54], but related abiotic conditions such as hypolimnetic volume will likely decrease as thermocline depth increases [2,55]. Primary producers, which form the trophic base of food webs in these lakes, are likely to be strongly influenced by warming temperatures. Potential consequences include increases in frequency and severity of harmful algal blooms [17,56], and earlier spring phytoplankton blooms [57]. In the SRM, shifts to earlier ice-off dates are increasing chorophyll *a* values (an indicator of phytoplankton production), suggesting that this thermal metric is an important driver of SRM lake primary production [36]. Climate change induced alterations of phytoplankton dynamics [58] can also lead to decreases in hypolimnetic dissolved oxygen concentration [18,59] and, consequently, reduced densities of benthic invertebrates [60]. Pelagic invertebrates are also influenced by warming thermal conditions of lakes through changes in zooplankton phenology [16] and increases in zooplankton productivity [61,62]. Climate driven thermal changes in SRM lakes may interact synergistically with elevated rates of atmospheric nitrogen deposition in high-elevation landscapes [63,64], with the potential to alter nutrient dynamics [65,66] in these normally oligotrophic systems, pushing them toward mesotrophic or eutrophic states [67].

High-elevation lakes, like those in the SRM, provide critical fish habitat, and changes to thermal characteristics are likely to alter fish life histories. Although it is unknown how many of these lakes were naturally fishless, owing to extensive stocking of native and non-native fishes initiated around the turn of the 20^th^ century [68,69], it is likely that some of the lakes in our analysis were fishless. Of the 27 lakes in our analysis, 23 currently have fish populations. One critical life history event for fish in high-elevation lakes is the movement between lakes and connecting streams for spawning. The phenology of spawning migrations can be shifted earlier for spring spawning fish [70] and later for fall spawning fish [71] when adult fish are exposed to increasing temperature in lake-stream networks. Earlier spawning events can result in a longer growing season for young-of-the-year fish. However, the growth and survival of young fish is a function of both temperature and prey availability [20,72]. Shifts in the timing of spawning events could result in a trophic mismatch of prey availability and demand during early life history events for fish [73,74], but such a phenomenon requires heterogeneity in seasonal warming rates [75]. The differences in warming rates between high-elevation SRM streams [31] and those reported for SRM lakes in this study indicate heterogeneity in warming rates and therefore, the potential for climate driven trophic mismatches during early fish life history. Paradoxically, the growth potential of adult fish in SRM high-elevation lakes may actually increase because these systems are currently quite cold, but this will vary by species and also be dictated by how prey (i.e., lower trophic levels) respond to these changing thermal conditions.

Although our results indicate a warming trend in overall thermal regimes of high-elevation SRM lakes, our study has potential limitations. First, the rates of change we present are based on climate projections using a medium to high emission scenario, which may be higher than those predicted under lower emissions. Using multiple emission scenarios would not influence the direction of these changes, but could alter the magnitudes of rates of change that our models predicted. Second, our focus on lake surface temperature does not address potential changes to other limnological features such as water column stratification and thermocline depth. Third, collection of more detailed data on drivers of ice cover phenology (e.g., timing and amount of snowfall or rain; [36]) could help refine predictions of ice dynamics in mountain lakes. Likewise, gathering basic physiographic data for lakes such as detailed bathymetry and continuous vertical thermal profiles would allow future studies of mountain lake temperature regimes to include more detailed processes such as water residence time, timing of runoff, and mixing dynamics (sensu [2,51]). These more detailed processes are particularly important to ice-off timing in SRM lakes which is likely driven by air temperature along with the timing and amount of snowfall or rain [36]. Finally, there are potential biases in the SNOTEL air temperature data caused by changes in sensor type and placement at SNOTEL sites, which mainly influenced daily maximum and minimum air temperature values [76]. This bias was strongest for minimum temperatures (warmer), whereas the bias in maximum temperature was weaker and varied by season (cooler in summer and warmer in winter). For our analysis we used SNOTEL daily mean air temperature, which was not addressed in the previously mentioned bias analysis [76]. We suggest that this bias is likely small, possibly influencing the magnitude but not the direction of these trends.

Although our results predict striking changes in the thermal regime of SRM high-elevation lakes, data were available for a relatively small sample of lakes arrayed in two fairly restricted regions of the SRM in Colorado. Thus, it is unlikely that we have characterized the full range of lake thermal response across the SRM landscape. Given the affordability and availability of commercial temperature loggers, the scope of future studies could be easily expanded to include collection of continuous lake temperatures. Additionally, new lake temperature data could easily be added to our predictive model approach (sensu [38]) to further explore the influence of climate change on high-elevation lakes. Ideally, a more comprehensive database of continuous temperatures could be used to create models for mountain lakes similar to the useful resources already available for mountain stream networks (e.g., NorWeST; http://www.fs.fed.us/rm/boise/AWAE/projects/NorWeST.html). Mountain lakes are important headwater sources of relatively pristine habitat for ecological communities and water for human uses, so understanding how changing climatic conditions are influencing these lakes is paramount to creating climate-smart management strategies for water resources and the biota that inhabit them.

## Supporting information

S1 Table Physio-chemical characteristics of the southern Rocky Mountain lakes in this analysis. Hydrogeomorphic characteristics of the 27 lakes with continuous records of lake surface temperature. Mean weekly air temperatures from SNOTEL sites were also used with our models of lake surface temperature to calculate the current (2004–2014) thermal conditions (M30AT).
(DOCX)

S2 Table Lake surface temperature model parameters and fit. Summary of lake temperature data (period of record) used to parameterize models of surface temperature and the parameter values for lake surface temperature models* (sensu [38]). The Nash-Sutcliff Coefficient (NSC; 1 = perfect fit) is a measure of model fit for non-linear logistic regression models [38].
(DOCX)

S1 File Daily mean lake surface water temperatures. Water temperatures (°C) collected from each of the 27 SRM lakes, summarized as daily mean lake surface water temperature. These water temperature data were used with air temperature from the closest SNOTEL station to fit models of daily lake surface temperature [sensu 38].
(CSV)

S2 File Mean weekly air temperature values from the closest SNOTEL station for each study lake. The mean weekly air temperature (°C) values from the closest SNOTEL station to each of 27 SRM lakes. This included both observed data (1998–2013) and monthly corrected projections from downscaled regional climate models (2035–2045, 2075–2085; [41]). These observed data were used to fit models of daily mean lake surface temperature. Both the observed and projected air temperature data were also used to reconstruct and forecast daily mean lake surface temperature.
(CSV)

S3 File Annual values for the four lake thermal metrics from each study lake. The annual values of the four lake surface temperature metrics (°C) used to summarize the response of each of the 27 SRM lakes to changes climatic conditions. These four metrics include meant annual lake surface temperature (MAT), mean summer lake surface temperature (MST), number of ice-free days (IFD), and maximum 30-day mean summer lake surface temperature (M30AT).
(CSV)
