# Supplementary material for: Thermal regimes of Rocky Mountain lakes warm with climate change
Source: PLoS One. 2017 Jul 6;12(7):e0179498. doi: 10.1371/journal.pone.0179498 (PMC5500263; doi:10.1371/journal.pone.0179498)
Supplement: S1 Table — Hydrogeomorphic characteristics of the 27 lakes with continuous records of lake surface temperature. Mean weekly air temperatures from SNOTEL sites were also used with our models of lake surface temperature to calculate the current (2004–2014) thermal conditions (M30AT). (DOCX) [file pone.0179498.s001.docx]

**S1 Table.** Hydrogeomorphic characteristics of the 27 lakes with continuous records of lake surface temperature. Mean weekly air temperatures from SNOTEL sites were also used with our models of lake surface temperature to calculate the current (2004-2014) thermal conditions (M30AT).

| **Lake** | **Surface area (km^2^)** | **Cumulative drainage area (km^2^)** | **Maximum depth (m)** | ***Elevation (m)** | **M30AT current** |
| --- | --- | --- | --- | --- | --- |
| Adams Lake | 0.02 | 00.77 | 05.8 | 3413 | 11.9 |
| Arrowhead Lake | 0.15 | 03.30 | 7.9 | 3408 | 07.4 |
| Bear Lake | 0.03 | 00.68 | 10.1 | 2915 | 09.7 |
| Big Cow Lake | 0.01 | 00.08 | 09.8 | 3463 | 09.6 |
| Bluebird Lake | 0.10 | 02.99 | 15.8 | 3338 | 09.6 |
| Boundary Lake | 0.01 | 00.78 | 06.0 | 3316 | 10.6 |
| Caddis Lake | 0.01 | 03.67 | 01.8 | 3277 | 08.9 |
| Crystal Lake | 0.11 | 00.86 | 38.1 | 3495 | 09.8 |
| Dream Lake | 0.01 | 02.25 | 04.3 | 2931 | 12.8 |
| Fern Lake | 0.03 | 05.58 | 09.5 | 2907 | 11.9 |
| Gem Lake | 0.02 | 00.09 | 07.6 | 3426 | 10.7 |
| Jewel Lake | 0.02 | 00.12 | 13.7 | 3412 | 10.6 |
| Lake Husted | 0.05 | 00.39 | 08.3 | 3384 | 12.1 |
| Lake Louise | 0.03 | 01.22 | 06.0 | 3378 | 10.0 |
| Lake Nanita | 0.13 | 02.69 | 17.1 | 3270 | 13.3 |
| Little Cow Lake | 0.01 | 00.03 | 06.1 | 3467 | 10.0 |
| Lost Lake | 0.03 | 01.17 | 06.4 | 3250 | 13.1 |
| Lower Hutcheson Lake | 0.02 | 03.97 | 06.7 | 3323 | 10.0 |
| Odessa Lake | 0.04 | 04.12 | 06.7 | 2931 | 09.9 |
| Pear Lake | 0.07 | 01.33 | 17.1 | 3229 | 13.4 |
| Pettingell Lake | 0.04 | 00.99 | 09.5 | 3213 | 15.7 |
| Sandbeach Lake | 0.05 | 00.65 | 07.6 | 3148 | 15.3 |
| Spruce Lake | 0.02 | 01.24 | 01.2 | 2947 | 15.6 |
| Timber Lake | 0.03 | 01.39 | 07.6 | 3370 | 12.6 |
| Trappers lake | 1.31 | 50.10 | 54.9 | 2935 | 14.7 |
| Upper Hutcheson Lake | 0.03 | 02.68 | 04.3 | 3413 | 09.2 |
| Ypsilon Lake | 0.04 | 03.41 | 16.8 | 3233 | 11.1 |
| *National Elevation Vertical Datum of 1988 (NAVD 88) | | | |  |  |
