# Supplementary material for: Thermal regimes of Rocky Mountain lakes warm with climate change
Source: PLoS One. 2017 Jul 6;12(7):e0179498. doi: 10.1371/journal.pone.0179498 (PMC5500263; doi:10.1371/journal.pone.0179498)
Supplement: S2 Table — Summary of lake temperature data (period of record) used to parameterize models of surface temperature and the parameter values for lake surface temperature models* (sensu [38]). The Nash-Sutcliff Coefficient (NSC; 1 = perfect fit) is a measure of model fit for non-linear logistic regression models [38]. (DOCX) [file pone.0179498.s002.docx]

**S2 Table.** **Lake surface temperature model parameters and fit.**

Summary of lake temperature data (period of record) used to parameterize models of daily mean lake surface temperature (*T_surface_*; Supporting Information S1 file) and the parameter values for lake surface temperature models* [sensu 38]. These models were fit using Mean weekly air temperature (*T_air_)* calculated from daily mean air temperature (Supporting Information S2 file) and *Tsurface* values. The Nash-Sutcliff Coefficient (NSC; 1=perfect fit) is a measure of model fit for non-linear logistic regression models [38].

| **Lake** | **Data years** | **Model parameters*** | | | | **NSC** |
| --- | --- | --- | --- | --- | --- | --- |
|  |  | Temp. max $(\alpha)$ | Temp. min ($\mu)$ | $\gamma$ | β |  |
| Adams Lake | 2001-2007, | 21.93 | 0 | 0.256 | 12.165 | 0.74 |
| Arrowhead Lake | 2002-2012, | 16.23 | 0 | 0.268 | 11.392 | 0.68 |
| Bear Lake | 2001-2012, | 16.68 | 0 | 0.191 | 13.394 | 0.88 |
| Big Cow Lake | 2012-2014, | 18.70 | 0 | 0.301 | 14.110 | 0.83 |
| Bluebird Lake | 2002-2008, | 15.95 | 0 | 0.262 | 11.118 | 0.78 |
| Boundary Lake | 2001-2005, | 19.62 | 0 | 0.236 | 12.088 | 0.70 |
| Caddis Lake | 2002-2010, | 20.63 | 0 | 0.244 | 11.706 | 0.85 |
| Crystal Lake | 2000-2011, | 17.90 | 0 | 0.296 | 9.828 | 0.81 |
| Dream Lake | 2001-2012, | 19.61 | 0 | 0.242 | 11.879 | 0.88 |
| Fern Lake | 2000-2012, | 19.47 | 0 | 0.213 | 12.372 | 0.84 |
| Gem Lake | 2012-2014, | 18.15 | 0 | 0.286 | 12.861 | 0.79 |
| Jewel Lake | 2012-2014, | 20.78 | 0 | 0.317 | 14.130 | 0.84 |
| Lake Husted | 2000-2012, | 22.96 | 0 | 0.253 | 11.598 | 0.89 |
| Lake Louise | 2000-2012, | 18.86 | 0 | 0.265 | 11.544 | 0.86 |
| Lake Nanita | 2001-2006, | 23.08 | 0 | 0.206 | 13.543 | 0.72 |
| Little Cow Lake | 2012-2014, | 18.95 | 0 | 0.308 | 13.824 | 0.81 |
| Lost Lake | 2001-2012, | 21.30 | 0 | 0.250 | 10.150 | 0.91 |
| Lower Hutcheson Lake | 2002-2009, | 18.48 | 0 | 0.263 | 12.138 | 0.84 |
| Odessa Lake | 2000-2010, | 16.63 | 0 | 0.218 | 12.883 | 0.83 |
| Pear Lake | 2002-2008, | 22.13 | 0 | 0.250 | 10.997 | 0.90 |
| Pettingell Lake | 2001-2002, | 18.95 | 0 | 0.273 | 9.084 | 0.90 |
| Sandbeach Lake | 2002-2010, | 24.37 | 0 | 0.259 | 10.722 | 0.89 |
| Spruce Lake | 2002-2012, | 28.82 | 0 | 0.198 | 14.167 | 0.90 |
| Timber Lake | 2001-2010, | 23.55 | 0 | 0.283 | 12.590 | 0.82 |
| Trappers lake | 2006-2008, 2011-2014, | 22.33 | 0.54 | 0.194 | 10.897 | 0.88 |
| Upper Hutcheson Lake | 2002-2009, | 18.31 | 0 | 0.238 | 12.797 | 0.82 |
| Ypsilon Lake | 2002-2012, | 19.06 | 0 | 0.259 | 9.158 | 0.87 |

$$*T_{surface}=\mu+\frac{\alpha-\mu}{1+e^{\gamma(\beta-T_{air})}}$$
